# ASO Author Reflections: The Hidden Burden: Impact of Allostatic Load on Colorectal Cancer Surgery Outcomes

**DOI:** 10.1245/s10434-025-17754-3

**Published:** 2025-07-03

**Authors:** Mujtaba Khalil, Timothy M. Pawlik

**Affiliations:** Department of Surgery, The Ohio State University, Wexner Medical Center and James Comprehensive Cancer Center, Columbus, OH USA

## Past

While disparities in surgical outcomes among socioeconomically disadvantaged individuals are well documented, the underlying biological mechanisms remain poorly defined.^1^ Individuals living in socially vulnerable neighborhoods are frequently exposed to chronic stressors such as financial insecurity, limited access to healthcare, and substandard living conditions, all of which contribute to physiological wear and tear over time.^1^ Allostatic load (AL), a composite index of multisystem physiologic dysregulation, captures the cumulative burden of chronic stress.^2^ Prior studies have demonstrated associations between AL and poor outcomes across various chronic diseases, yet its role in surgical populations remains underexplored.^2^ Therefore, the current study aims to characterize the relationship between AL, social vulnerability, and postoperative outcomes following colorectal cancer (CRC) surgery.^3^

## Present

Among 40,520 individuals, mean patient age was 67.7 years (SD ± 13.9), roughly half of the patients were male (*n *= 20,573; 50.8%), and Charlson Comorbidity Index score was high (CCI > 2; *n *= 33,132; 81.8%). Overall, 7.1% (*n *= 2897) of the patients had a high AL. Notably, AL increased with increasing SVI (ref: low; medium: 1.10 [95% CI 1.01–1.20]; high: 1.17 [95% CI 1.07–1.28]). High AL was associated with a 48% increased risk of postoperative complications (OR 1.48; 95% CI 1.38–1.58), a 79% increased risk of an extended length of stay (OR, 1.79; 95% CI 1.67–1.90), and a twofold (OR 2.13; 95% CI 1.90–2.37) increase in the risk of mortality within 30 days of surgery. 

## Future

As health systems increasingly focus on addressing social determinants of health, our findings highlight the importance of integrating biological markers of chronic stress—such as AL—into perioperative risk assessment.^3^ Incorporating AL into preoperative evaluation may help identify patients at elevated risk for complications, extended hospitalizations, and early postoperative mortality.^4^ Since AL is calculated using routinely collected clinical data, it can be feasibly implemented in surgical workflows to enhance risk stratification.^5^ Moving forward, prospective studies are needed to determine whether interventions such as prehabilitation, stress-reduction programs, or enhanced perioperative support can mitigate AL and improve outcomes in high-risk populations.^3^ Additionally, longitudinal assessments of AL could help distinguish between reversible physiologic stress and more permanent biologic dysfunction.^5^ Ultimately, targeting AL may represent a novel strategy to reduce disparities and improve surgical equity for patients with colorectal cancer.^4^
